# Post-Discectomy Infection: A Critical Review and Suggestion of a Management Algorithm

**DOI:** 10.3390/jcm13051478

**Published:** 2024-03-04

**Authors:** Constantinos Chaniotakis, Christos Koutserimpas, Andreas G. Tsantes, Dimitrios V. Papadopoulos, Christothea-Alexandra Tsiridis, Apostolos Karantanas, Kalliopi Alpantaki, Alexander Hadjipavlou

**Affiliations:** 1 Department of Orthopaedics and Trauma Surgery, “Venizeleion” General Hospital of Heraklion, 71409 Crete, Greece; kostas_chanio1994@hotmail.com (C.C.); apopaki@yahoo.gr (K.A.); 2 Department of Orthopaedics and Traumatology, “251” Hellenic Air Force General Hospital of Athens, 11525 Athens, Greece; 3 Department of Anatomy, School of Medicine, National and Kapodistrian University of Athens, 11527 Athens, Greece; 4 Laboratory of Haematology and Blood Bank Unit, Attikon Hospital, School of Medicine, National and Kapodistrian University of Athens, 12462 Athens, Greece; andreas.tsantes@yahoo.com; 5 Microbiology Department, Saint Savvas Oncology Hospital, 11522 Athens, Greece; 6 Second Department of Orthopaedics, School of Medicine, National and Kapodistrian University of Athens, 14233 Athens, Greece; di_papadopoulos@yahoo.gr; 7 Department of Medicine, St. George’s University of London, London SW17 0RE, UK; theatsiridis@gmail.com; 8 Department of Medical Imaging, University Hospital, 71110 Heraklion, Greece; akarantanas@gmail.com; 9 Department of Orthopaedic Surgery and Rehabilitation, University of Texas Medical Branch, Galveston, TX 77550, USA; ahadjipa@yahoo.com

**Keywords:** post-discectomy complications, spine surgery, osseous infections, treatment algorithm, spinal infection

## Abstract

Postoperative discitis (POD) accounts for 20% to 30% of all cases of pyogenic spondylodiscitis, while POD may be mis-or-under-diagnosed, due to the vague related symptomatology and the non-specific imaging findings. Most studies report infection rate of less than 1%, which increases with the addition of non-instrumented fusion to 2.4% to 6.2%. It remains controversial whether POD is caused by an aseptic or infectious process. Positive cultures are presented only in 42–73% of patients with *Staphylococcus* species being the most common invading organisms, while *Staphylococcus aureus* is isolated in almost 50% of cases. The onset of POD symptoms usually occurs at 2–4 weeks after an apparently uneventful operation. Back pain and muscle spasms are usually refractory to bed rest and analgesics. Magnetic Resonance Imaging (MRI) is the most sensitive and specific imaging diagnostic technique. Antimicrobial therapy depends on the results of tissue cultures, and along with bracing represents the mainstay of management. Surgical intervention is necessary in patients failing conservative treatment. For the majority of cases, extensive surgical debridement, antibiotic therapy, and orthosis immobilization are effective in eliminating the infection. According to this, we recommend an Algorithmic approach for the management of POD. Postoperative infections after spinal surgery pose a certain clinical challenge, and in most cases can be treated conservatively. Nevertheless, disability may be persistent, and surgery could be necessary. The purpose of this concise review is to describe the manifestation of post-discectomy infection, its pathogenesis and particularly a rational approach for its management.

## 1. Introduction

The clinical entity known as postoperative discitis (POD) was initially described by Frank Turnbull in the early 90s. It represents approximately 20% to 30% of all instances of pyogenic spondylodiscitis [1,2,3,4,5]. Discitis or spondylodiscitis can lead to serious consequences; hence, prompt treatment is imperative. Regrettably, POD might go undetected or be diagnosed too late due to misinterpretation of postoperative clinical and imaging presentations [6].

Most reports indicate infection rates of less than 1% (Table 1), which increase with the addition of fusion (without instrumentation), reaching up to 6.2% [1,7,8]. The incidence of infection after instrumented fusion is even higher, estimated up to 20% [1]. Furthermore, the impact of intraoperative use of a microscope on the rate of postoperative spondylodiscitis is not clear. Some authors have demonstrated a negative impact with up to 5% increase of the infection rate [9,10], while others have reported positive impact with reduction of the infection rate from 2.8% to 0.4% [11].

Minimally invasive surgeries, like percutaneous discectomy, appear to have a lower incidence of infection. The incidence according to Bonaldi et al. is 0.26% [20]. Kang TW et al. in a retrospective nationwide cohort study of patients undergoing percutaneous endoscopic lumbar discectomy in Korea reported infection rate of 0.83% [21]. A more recent article by Mahan MA in a retrospective multicenter cohort study of a total of 1277 endoscopic discectomies reported infection’s incidence 0.001% [22]. These infections usually resolve without any clinical or radiological sequelae [20].

The reported rate of disc space infections following other diagnostic or therapeutic procedures, such as discography or chemonucleolysis, stands at 2.3% [23,24]. The incidence of POD seems to be related to the extent of tissue damage that occurs during the surgical procedure [1].

The purpose of this critical review is to provide valuable insights into POD, describing the manifestation of this infection, its pathogenesis, and a rational approach to its management. Furthermore, an algorithm for the management of such infections is described and proposed.

## 2. Methods

In an effort to better understand and evaluate infections following discectomies, a critical review of the available literature was performed through October 2023 using the MEDLINE and the PubMed databases. PRISMA guidelines were not applicable, since this is a critical-narrative review. The available literature was summarized and evidence on pathogenesis, clinical manifestation, imaging modalities for diagnosis, and management of these infections was critically reviewed and presented.

Furthermore, it should be noted that we also describe and suggest a treatment algorithm for such infections that is illustrated and analyzed.

## 3. Results

### 3.1. Pathogenesis

POD is believed to occur due to direct inoculation of the avascular disc space during surgery, likely by the skin flora or environmental factors [25]. A different route of infection can be also through continuous spread during the early postoperative period from adjacent retrodiscal tissue [6]. A third source of infection is through hematogenous dissemination [26].

Injury to the disc or vascular compromise during surgery may result in the so-called aseptic discitis [27], although this entity has been seriously challenged. Fraser et al., based on animal experiments, propose that after about a 6-week period, a disc infected with *Staphylococcus aureus* may transition to an “aseptic” state [28]. They illustrated that vascular granulation tissue from the subchondral bone invades, absorbs, and manages the infectious process. This mechanism could elucidate the relatively mild course observed in some cases of spondylodiscitis. Table 2 summarizes the spread type of the infection.

It is widely recognized that primary hematogenous pyogenic spondylodiscitis can sometimes progress to epidural abscess and sepsis, resulting in severe consequences, including death [29]. Nevertheless, secondary post-discectomy infection typically follows a milder course compared to primary hematogenous spondylodiscitis, which is associated with higher risk factors [29].

It remains controversial whether POD is caused by an aseptic or infectious process since positive cultures are detected in only 42–73% of patients [25]. Injury to the end plate, hematoma formation, and necrotic tissue during surgical procedure provide ideal conditions for bacterial growth. POD is a mono-bacterial infection in most cases, with *Staphylococcus* being the predominant primary pathogen [25,30]. Specifically, *Staphylococcus aureus* is isolated in almost 50% of cases. Other common Gram-positive species include *Staphylococcus epidermidis* and other coagulase-negative *Staphylococcus* species. Common Gram-negative organisms, such as *Escherichia coli*, *Pseudomonas aeruginosa*, *Klebsiella pneumoniae*, *Enterobacter cloacae*, *Bacteroides* and *Proteus* species, have been also isolated from infected surgical sites [25,30].

Pull ter Gunne et al. presented the results of bacteria cultures in patients with PODs. *Staphylococcus aureus* was the most common organism (65.1%), while *Enterococcus faecalis* and *Escherichia coli* were presented in 14.5% and 10.8%, respectively [31]. Many studies have also reported the presence of fungal infections (*Candida* and *Aspergillus fumigatus*). The most precise cultures are those acquired during surgical debridement performed prior to the initiation of antimicrobial agents [31,32].

It has been suggested that lumbar spine spondylodiscitis has lower incidence of neurological deficits, with the exception of POD caused by *Serratia marcescens*, as compared to more cephalad involvement [29]. *Serratia marcescens*, which typically resides saprophytically in water, soil, and the human alimentary tract, has been commonly associated with nosocomial infections affecting the lungs, meninges, urinary tract, injured tissue, and occasionally, bones and joints [33,34]. This infection carries a high mortality rate ranging between 25% and 52%, particularly when accompanied by bacteremia. Although uncommon, spine infection post-surgery has been reported to lead to spondylodiscitis with purulent epidural abscess. Such cases are characterized by an intense clinical presentation within a week after surgery and may be complicated by paralysis [35,36,37,38]. Eventually, discitis is expected to result in bony or fibrous ankylosis of the adjoining vertebrae. At this stage, the patient becomes completely asymptomatic. However, pain may persist if the fibrous union does not become solid. Occasionally bone destruction may occur bringing about painful kyphotic deformity.

Infections following diagnostic or therapeutic interventional spinal injections, such as intradiscal injections, epidural steroid injections (ESI), facet joint injections, and discography are reported to range from 1% to 2% [39,40,41]. Table 3 presents the incidence of infections among different types of these procedures. Fungal (*Exserohilum rostratum*, *Aspergillus fumigatus*), viral (varicella-zoster virus, HSV-1, and HSV-2), and bacterial (methicillin-resistant *Staphylococcus aureus*, methicillin-resistant *Staphylococcus epidermidis*) infections following ESI have been documented in limited studies [42,43,44,45]. In a retrospective review of 11,980 facet joint injections, Kim et al. presented 8 cases of infections, including 1 case of systemic fungal infection (*Aspergillus*) that spread to the spine and 7 cases of infectious spondylitis [46]. Infections after intradiscal injections are the most common among types of interventional spine procedures [39]. Bosnak et al. mentioned 10 cases of nosocomial spondylodiscitis with *Pseudomonas aeruginosa* after intradiscal electrothermal therapy [47]. Additionally, infections after intradiscal injections of Cytokine Blockers (tocilizumab, interleukin-6 receptor antibody, and etanercept) have been reported in three studies, with an incidence of 2.11% (Table 3) [39].

### 3.2. Clinical Manifestation

The onset of POD symptoms usually occurs at 2–4 weeks (ranges 1–6 weeks) after an otherwise uneventful operation [18,48,49,50,51,52]. The presenting signs and symptoms include acute onset of severe and continuous back pain (88%), muscle spasms (76.4%), stiffness, while sciatica and positive Lasegue’s sign are present in 87% of cases, and pseudo-Gower sign in 73% of them [3,27,53,54]. Pain and muscle spasms are usually refractory to bed rest and analgesics [52]. At the incision site, there is frequently erythema, warmth, and often drainage of fluid. Fever may be present in 11–68% of all cases. In contrast to primary hematogenous pyogenic infection, no mortality rates have been observed by most studies, except one cohort that reported 1.4% mortality rate [29,55].

### 3.3. Laboratory Investigation

At the time of diagnosis, the average erythrocyte sedimentation rate (ESR) is approximately 60 mm/h [18,25,26,53,56,57,58,59,60,61,62,63,64]. Among patients who underwent an uncomplicated discectomy, 97% exhibited c-reactive protein (CRP) values below 2.5 μg/mL, ESR values below 45 mm/h, temperatures equal to or below 37.5 °C, and normal magnetic resonance imaging (MRI) findings within the initial 10 days post-surgery. Consequently, deviations from these values could indicate a potential red flag for POD [6]. Persistent elevated ESR and CRP along with typical MRI findings suggest POD. It is known that CRP typically lessens roughly 10 days postoperatively. Therefore, patients with unexpected rise in CRP beyond 2 weeks after the procedure should be investigated for POD [49]. Some authors suggest that CRP is the most sensitive indicator of POD [30,57]. In a cohort of 18 patients presented with clinical features of POD, ESR and CRP were elevated in 88% and 81% of cases respectively; and MRI showed typical florid inflammation and granulation tissue with low signal intensity in T1 images and high signal intensity in T2-weighted images in 60% cases [25].

The white blood cell (WBC) count is a poor marker for surgical site infection in POD. The WBC value fluctuations depend on the host immune system and the type of pathogen. Less than 50% of cases of postoperative spinal infections will display an elevated WBC [58]. Additionally to the standard inflammatory markers, novel laboratory tests have been employed to trace postoperative spinal infections. Serum amyloid A (SAA) decreases rapidly postoperatively after its peak on day 3, which enables SAA to represent a great early inflammatory indicator in the investigation of early postoperative infection when the CRP and ESR will still be elevated due to the procedure. Moreover, SAA is not affected by corticosteroid administration, which is commonly given after spine surgery and may affect other inflammatory markers. Presepsin has also been tested as an early inflammatory marker after spinal surgery [58]. Both assays are still under evaluation and they have not replaced traditional markers yet in the evaluation of POD.

Blood cultures should be obtained from 2 sites prior to initiating antibiotics, even though they will be positive in less than 50% of the cases. Also, investigation of any suspicious extraspinal source of infection should be performed. If there is evidence of urinary or respiratory trunk infection urine and sputum samples should be sent for cultures respectively [58]. As already mentioned direct inoculation may be the commonest source of POD; however, hematogenous spread is also important [25] (Table 2).

The most valuable laboratory method for diagnosing POD is percutaneously CT-guided biopsy. Samples (disc material, subchondral bone and abscess fluid) are collected from the affected disc space as well as the surrounding inflammatory soft tissue. Fluoroscopic guide percutaneous transpedicular biopsy offers similar accuracy and adequacy and can be used in cases that CT-guided biopsy is not available [59]. In a recent systematic review aiming to determine the diagnostic culture yield of CT-guided biopsies performed in cases of suspected spinal infections, the authors reported only 33% diagnostic accuracy of CT-guided biopsies, in terms of positive cultures. In most instances, a causative organism is not identified. Enhancements in biopsy techniques and specimen handling, such as immediately transferred and cultivated specimens, have been described in order to improve culture yield. Repeated biopsy may be necessary to establish the diagnosis [16]. Open biopsy can increase the diagnostic culture yield but it is recommended in patients with severe infections, requiring surgical management [61,62]. Fungal and mycobacterium infection diagnosis, although extremely rare, should be considered especially when cultures from the biopsy site are negative for bacteria and the symptoms insist despite antibiotic treatment. If the diagnosis is strongly suspected surgical biopsy is recommended [63,64].

Molecular medicine techniques such as nucleic acid amplification testing (NAAT) have been proposed in order to increase accuracy in cases of negative aerobic and anaerobic cultures or in patients who have already taken antibiotics [65].

### 3.4. Imaging

**Figure 1:**
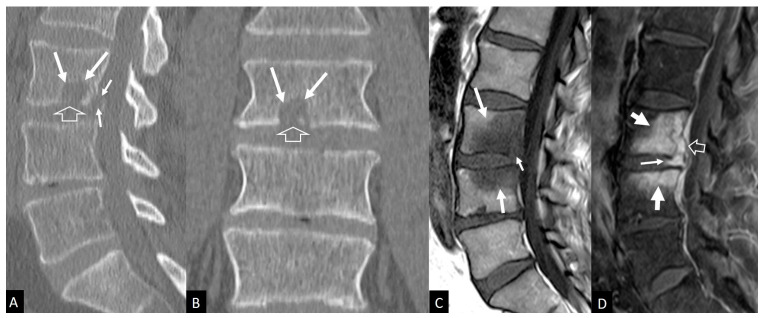
*Staphylococcus aureus* spondylodiscitis in a 53-year-old male, with a history of microdiscectomy at the L3-L4 level, 6w prior to imaging. Sagittal (**A**) and coronal (**B**) CT reformatted images showing osteolysis in the lower L3 vertebral body (arrows) with cortical disruption of the lower epiphyseal plate (open arrows). An additional osteolysis with cortical disruption is shown posteriorly (short arrows). (**C**) Sagittal T1-w MR image showing bone marrow edema in the vertebral bodies L3 and L4 (arrows) and cortical disruption of the posterior epiphyseal plate (short arrow). (**D**) Fat suppressed contrast-enhanced sagittal MR image showing enhancement of the bone marrow (thick arrows) and the posterior disc (thin arrow). Intravertebral abscess formation is shown posteriorly (open arrow).

**Figure 2:**
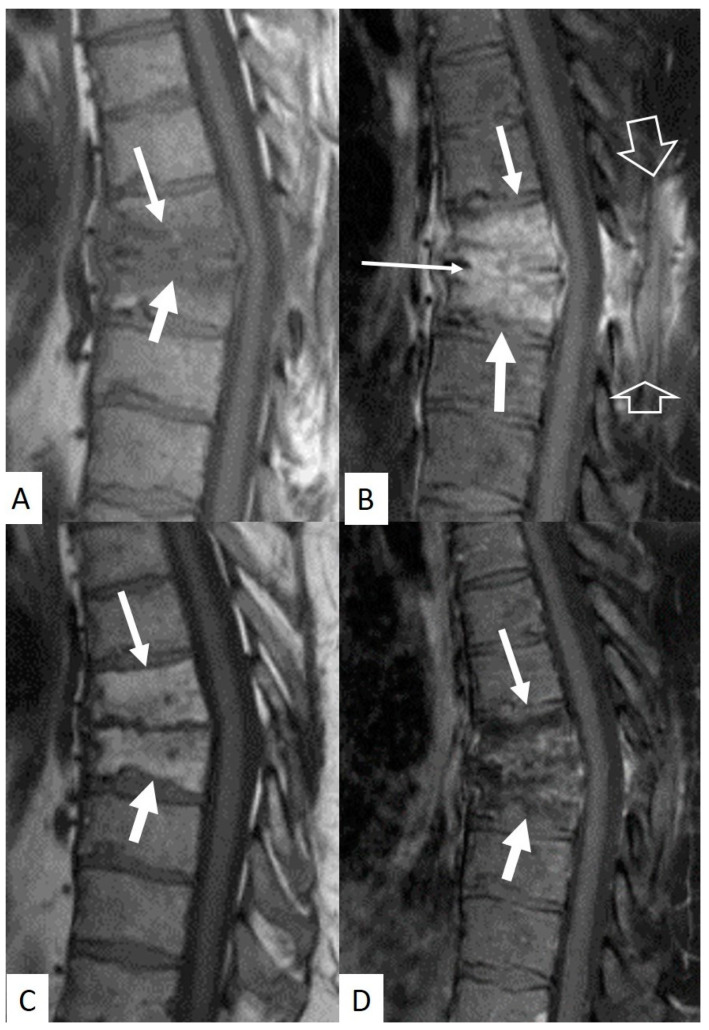
A 47-year-old male patient with *Salmonella* spondylodiscitis following previous discectomy and hemilaminectomy, 8w prior to imaging. (**A**) Sagittal T1-w MR image, showing bone marrow edema (arrows). (**B**) Sagittal fat suppressed contrast-enhanced MR image showing enhancement of the bone marrow lesions (arrows), enhancement of the intervertebral disc T7–T8 (horizontal thin arrow), mild kyphosis and normal postoperative changes posteriorly (open arrows). Sagittal T1-w (**C**) and fat suppressed contrast-enhanced T1-w (**D**) MR images 20 weeks after initiation of antibiotic treatment, show fatty metaplasia in the bone marrow of the T7 and T8 vertebral bodies without any abnormal enhancement, in keeping with healing (arrows). Note resolution of the postoperative findings.

**Figure 3:**
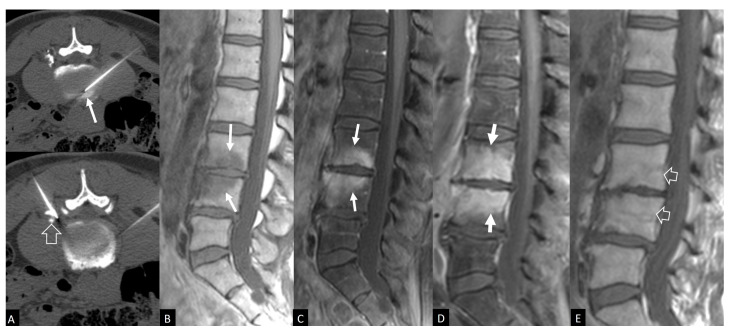
Spondylodiscitis in a 57-year-old female, 3w after CT-guided treatment with radiopaque gelified ethanol injection in the disc. (**A**) Axial CT images with the patient prone, show the needle placement within the L3-L4 disc and the injected material (arrow) and the needle placement close to the L3 exiting root-perineural injection with contrast (open arrow). Sagittal T1-w (**B**) and fat suppressed contrast-enhanced T1-w (**C**) MR images 3w after the procedure, show bone marrow edema (arrows). (**D**) Sagittal fat suppressed contrast-enhanced T1-w (**D**) MR image 5w after the procedure, shows deterioration of the bone marrow edema (arrows). (**E**) Sagittal T1-w MR image 8 months later, shows restoration of the normal bone marrow signal (open arrows) in keeping with complete resolution of symptoms. (Case courtesy: K. Spanakis).

Plain radiographic findings typically appear between 6 to 8 weeks postoperatively. Disc space narrowing is usually the first finding. Other signs are osteopenia, end plate erosion and kyphotic deformity. Computed Tomography (CT) scan, a more sensitive tool, may demonstrate end plate erosion, bone resorption and disc space narrowing at 3–6 weeks postoperatively (Figure 1) [25,27]. MRI is the most sensitive and specific imaging diagnostic technique (Figure 1, Figure 2 and Figure 3) [66,67]. The possibility of a painful recurrent or residual disc herniation can be ruled out with MRI [68]. The characteristic MRI findings are hypointense bone marrow lesions on T1-w images, and hypertense on T2-w images and fluid sensitive sequences, i.e., Short Tau Inversion Recovery (STIR) Gadolinium-enhanced T1-w MR images demonstrate enhancement of the bone marrow lesions and the intraosseous pus formation. A hyperintense signal surrounding the pus (Halo sign), indicates vascular inflammatory granulation tissue [29]. Complete resolution of the infectious process, is confirmed with restoration of the normal signal of the bone marrow on T1-w MR images with or without fatty metaplasia (Figure 2 and Figure 3) Radionuclide 67 Ga has excellent intra-rater and interrater reliability and reproducibility. Sequential 67 Ga scanning proves to be a dependable and sensitive method for assessing the response to antimicrobial treatment [29].

### 3.5. Management

*Staphylococcus aureus* represents the most commonly isolated organism in POD. Thus, empirical treatment should always include an anti-staphylococcal antimicrobial regimen. Nevertheless, attempts to isolate the causative organism should be made, in order to proceed to targeted antimicrobial treatment [53]. There is no agreement regarding the optimal utilization of antimicrobial agents in the treatment of postoperative spondylodiscitis. Intravenous (IV) antimicrobial agents that have been successfully used as a single regimen include tobramycin, cephazolin, clindamycin and cephalothin. Antimicrobials that have been used successfully in combination include methicillin, nafcillin, rifampin, cephazolin, penicillin, vancomycin and cephalothin. Singh et al. reported their experience in 31 patients treated for POD. The duration of IV antibiotic therapy was 6 weeks in responders. Additional 3 weeks of IV antibiotic treatment was given in patients who failed to improve with conservative treatment and were taken up for surgical debridement and fixation. A combination of three antibiotics was initially used in all patients. The most common antibiotics used were vancomycin/cefepime/linezolid along with amikacin and metronidazole. Antibiotic treatment was tailored in two culture positive patients after surgical debridement. Antifungal treatment (fluconazole 150 mg PO for 3 weeks) was added in one urine culture positive patient. Six weeks of oral antibiotic treatment (linezolid 600 mg OD + ciprofloxacin 500 mg BID) were given in all patients at the time of discharge [49]. In a recent cohort study of 75 patients with POD, Meropenem, Flucloxacillin, Linezolid and Fusidic Acid were effective against *Staphylococcus aureus* and *Staphylococcus epidermidis*. Additionally, *Escherichia coli*, *Enterobacter* species and *Pseudomonas aeruginosa* were sensitive to Ciprofloxacin and Tobramycin. Ceftriaxone (3rd-generation Cephalosporin) was used empirically and during primary surgery but was resistant to *Staphylococcus aureus*, *Staphylococcus epidermidis*, *Escherichia coli* in 40% cases [18]. Patients are commenced on IV antimicrobial agents for 4 to 6 weeks, followed by per os antibiotics for another 6 weeks [48,49,51,69]. With the early initiation of IV antibiotic treatment, the ESR typically decreases to normal levels within approximately 90 days [48]. With proper treatment, POD recurrence is uncommon, ranging from 0% to 4% [3,56]. The reported success rates with conservative antimicrobial treatment ranges from 35.5% to 77.8% [3,25].

Surgical intervention is necessary in patients failing conservative treatment. In the majority of cases, thorough surgical debridement, antibiotic therapy, and orthosis immobilization can effectively eliminate the infection [32]. Immobilization of the lumbar spine with orthotic devices is an essential component of the overall management. A meticulous surgical debridement must be performed, and the wound must be explored to remove all affected tissue. Samples from each tissue layer should be routinely sent for microbiological examination for aerobic, anaerobic, and fungal pathogens. Certain cases may necessitate even more extensive surgery [70]. The indications for surgery include severe destruction of endplates, abscess formation, chronic osteomyelitis with biomechanical instability, neurologic deficits, local kyphosis, severe pain, and pseudoarthrosis. The surgical plan encompasses, usually, posterior approach with combination of debridement and stabilization. Fusion may not be needed in all cases. The nature of the pain is highly suggestive of mechanical instability. Posterolateral fusion leads to spontaneous anterior interbody fusion, a process that is accelerated when disc space debridement is performed [70,71].

R. Santhanam et al. reported the results of a conservative regime in 18 patients with spondylodiscitis including strict bed rest and antimicrobial therapy (vancomycin, linezolid and cefaperazone with sulbactam). A total of 14 out of 18 patients (77.8%) improved with non-operative management. In those 4 patients, who did not respond to antibiotic treatment, surgical treatment was performed which consisted of irrigation and debridement with curettage of the disc space granulation tissue, followed by spinal fusion through transpedicular fixation (posterior approach) [25]. The authors reported that instrumentation could provide a more robust stability to the infected spine that could hasten the healing process [70].

Conservative treatment has been proven to be sufficient for the uncomplicated POD cases. However, a variable degree of disability due to pain may persist for 3–4 months.

Minimally invasive spine surgery may offer early pain relief in patients with POD and a higher diagnostic yield to targeted antibiotic treatment [72]. Early debridement through endoscopic transpedicular discectomy has demonstrated to expedite the natural healing process, halt progression to bone destruction, and prevent the formation of epidural abscesses [73]. The benefits of this minimally invasive approach include effective drainage of infected material, collection of adequate tissue samples for histological and microbiological analysis, implementation of a suction-irrigation system, rapid alleviation of pain and discomfort, and early mobilization of the patient. This procedure should be also considered as a cost-effective approach, since patient’s hospital stay that is necessary for bed rest and analgesia can be drastically curtailed to one or two days, as opposed to open surgical techniques in which several weeks may be needed [73].

Similar results have also been reported through percutaneous endoscopic discectomy [74,75,76]. Full endoscopic debridement and drainage have been employed to treat POD and psoas muscle abscesses even in elderly patients and patients with multiple medical comorbidities. The major advantage of this technique over traditional open surgery is that it can be performed even under local anesthesia [77,78]. Different surgical approaches can be used such as interlaminar, transforamina or bi-portal with success rates over 80% [77,78]. For anterior pathology, transforaminal discectomy and drainage is an optimal approach targeting anterior column directly without destructing posterior structure. While, posterior (interlaminar) approach is suitable for “posterior” epidural abscess or paraspinal abscess. In cases with both pathologies, both approaches could be used simultaneously [77,79]. Endoscopic drainage can be successfully combined with other minimally invasive surgical techniques with favorable results [80].

The overall reported success rates of these procedures, as indicated by level IV studies, vary from 76% to 87% [73,76]. A common factor contributing to the prompt healing in these procedures is the shaving or penetration of the subchondral plate of the affected intervertebral disc [81] so that vascular granulation tissue from the vertebral body can spread and be involved in the healing process.

## 4. Suggestion of a Treatment Algorithm

**Figure 4:**
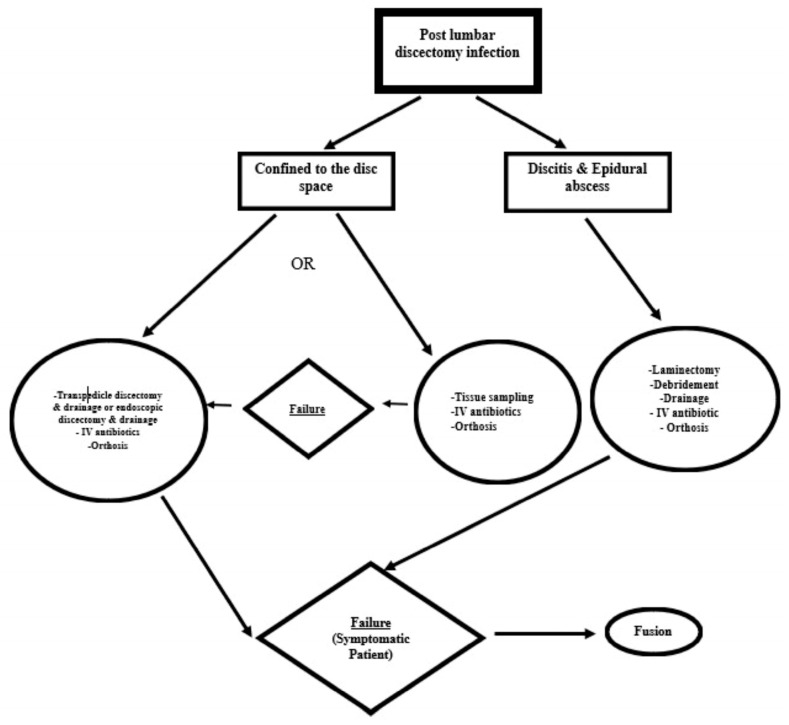
Algorithmic approach for management of post discectomy infection.

On the basis of available reports [6] for the management of hematogenous pyogenic spondylodiscitis [29,82,83] and postoperative infections [24], we recommend the following Algorithmic approach for the treatment of POD (Figure 4):

- If the infection is limited to the disc space, either conservative treatment or minimally invasive surgery may be chosen.
  - Conservative treatment consists of IV antimicrobial agents after appropriate sampling for tissue culture, and orthosis [6,12,14,15,29]. Antimicrobial agents are administrated IV for 4–6 weeks, followed by oral administration for another 6 weeks. The majority of available reports recommend some form of immobilization, such as bed rest until the patient achieves comfort, along with the use of orthotic devices [15,51,73,84]. We suggest employing rigid or semirigid orthosis until fibrous or bony ankylosis occurs. The usual time required is 3 months. In case of conservative treatment failure, surgical intervention is mandatory.
  - Minimally invasive surgical techniques, like transpedicular discectomy or endoscopic discectomy (through transforaminal-posterolateral or interlaminar-posterior approaches), along with drainage of purulent material, have demonstrated cost-effectiveness in managing primary hematogenous pyogenic discitis. This is particularly applicable when the condition is not complicated by serious neurological deficits or destructive bony lesions [29,85].
- In the event the infection extends to form a retrodiscal abscess (epidural abscess) or if conservative treatment fails, we recommend a more aggressive approach involving surgical drainage, debridement, and irrigation [6,29,73,85].
- Finally, we recommend interbody fusion (posterolateral) if non-operative treatment or surgical debridement fails [85].

## 5. Conclusions

Postoperative infections following spinal surgery pose a certain clinical challenge, and in most cases can be treated conservatively. A conservative protocol including targeted antimicrobial therapy based on the results of tissue cultures along with bracing is the mainstay of treatment. However, a variable degree of disability due to pain may persist for several months. These infections are considered healed when solid stabilization between the two adjoining vertebrae is achieved through solid fibrous unions or bony arthrodesis. Loose fibrous union or kyphosis from bone destruction may lead to chronic pain. Minimally invasive surgery by means of full-endoscopic discectomy may speed up the healing process and minimize the lengthy disability period, as opposed to open surgical techniques.

## Figures and Tables

**Table 1:** Infection Rates after conventional discectomy.

Infection Rate	Authors
3.0%	Pilgaard, 1969 [12]
3.1%	Wright, 1970 [13]
0.6%	Horwitz et al., 1975 [8]
0.8%	El-Gindi et al., 1976 [14]
0.75%	Lindholm and Phylkkanen, 1982 [15]
0.7%	Puranen et al., 1984 [16]
5.0%	Leung, 1988 [17]
0.6%	Heller et al., 1992 [9]
3.7%	Rohde et al., 1998 [18]
0.86%	Weinstein et al., 2000 [7]
1.3%	Hadjipavlou et al., 2005 [1]
2.6%	Rahman et al., 2008 [19]

**Table 2:** Pathogenesis types of Postoperative discitis.

Pathogenesis	
Types	Definition
Direct inoculation of the avascular disc space.	At time of surgery, by the skin flora or the environment.
Continuous spread.	During early postoperative period. From adjacent retrodiscal tissue.
Hematogenous dissemination.	Injury to the disc or vascular compromise. Vascular granulation tissue from the subchondral bone

**Table 3:** Infections following interventional spine procedures. ESI: epidural steroid injections.

Interventional Spine Procedures	
Type	Incidence
ESI	0–0.1%
Intradiscal injections	1.05%
Discography	0–4%
Facet joint injections	0.04%

## Data Availability

Not applicable.

## References

[1] Hadjipavlou A.G., Tzermiadianos M.N., Katonis P.G., Kontakis G.M., Szpalski M., Gunzburg R. (2005). How important is postoperative infection in the spine and what are the available therapeutic options?. The Failed Spine.

[2] Turnbull F. (1953). Postoperative inflammatory disease of lumbar discs. J. Neurosurg..

[3] Jiménez-Mejías M.E., Colmenero J.d.D., Sánchez-Lora F.J., Palomino-Nicás J., Reguera J.M., de la Heras J.G., García-Ordonez M.A., Pachon J. (1999). Postoperative spondylodiskitis: Etiology, clinical findings, prognosis, and comparison with nonoperative pyogenic spondylodiskitis. Clin. Infect. Dis..

[4] Bontoux D., Codello L., Debiais F., Lambert de Cursay G., Azais I., Alcalay M. (1992). Infectious spondylodiscitis. Analysis of a series of 105 cases. Rev. Rhum. Mal. Osteo-Articul..

[5] Meys E., Deprez X., Hautefeuille P., Flipo R.M., Duquesnoy B., Delcambre B. (1991). Role of iatrogenic spondylodiscitis among pyogenic spondylodiscitis. 136 cases observed between 1980 and 1989. Rev. Rhum. Mal. Osteo-Articul..

[6] Schulitz K.P., Assheuer J. (1994). Discitis after procedures on the intervertebral disc. Spine.

[7] Weinstein M.A., McCabe J.P., Cammisa F.P. (2000). Postoperative spinal wound infection: A review of 2391 consecutive index procedures. J. Spinal Disord..

[8] Horwitz N.H., Curtin J.A. (1975). Prophylactic antibiotics and wound infections following laminectomy for lumber disc herniation. J. Neurosurg..

[9] Heller J.G., Rothman R.H., Simeone F.A. (1992). Postoperative infections of the spine. The Spine.

[10] Kulkarni A.G., Patel R.S., Dutta S. (2016). Does Minimally Invasive Spine Surgery Minimize Surgical Site Infections?. Asian Spine J..

[11] Dauch W.A. (1986). Infection of the intervertebral space following conventional and microsurgical operation on the herniated lumbar intervertebral disc. A controlled clinical trial. Acta Neurochir..

[12] Pilgaard S. (1969). Discitis (closed space infection) following removal of lumbar inervertebral disc. J. Bone Jt. Surg. Am..

[13] Wright R.L. (1970). Septic Complications of Neurological Spinal Procedures.

[14] El-Gindi S., Aref S., Salama M., Andrew J. (1976). Infection of intervertebral discs after operation. J. Bone Jt. Surg. Br..

[15] Lindholm T.S., Pylkkänen P. (1982). Discitis following removal of intervertebral disc. Spine.

[16] Puranen J., Mäkelä J., Lähde S. (1984). Postoperative intervertebral discitis. Acta Orthop. Scand..

[17] Leung P.C. (1988). Complications in the first 40 cases of miscodiscectomy. Clin. Spine Surg..

[18] Piotrowski W.P., Krombholz M.A., Mühl B. (1994). Spondylodiscitis after lumbar disk surgery. Neurosurg. Rev..

[19] Rahman M., Summers L.E., Richter B., Mimran R.I., Jacob R.P. (2008). Comparison of techniques for decompressive lumbar laminectomy: The minimally invasive versus the “classic” open approach. Minim. Invasive Neurosurg..

[20] Bonaldi G., Belloni G., Prosetti D., Moschini L. (1991). Percutaneous discectomy using Onik’s method: 3 years’ experience. Neuroradiology.

[21] Kang T.W., Park S.Y., Oh H., Lee S.H., Park J.H., Suh S.W. (2021). Risk of reoperation and infection after percutaneous endoscopic lumbar discectomy and open lumbar discectomy: A nationwide population-based study. Bone Jt. J..

[22] Mahan M.A., Prasse T., Kim R.B., Sivakanthan S., Kelly K.A., Kashlan O.N., Bredow J., Eysel P., Wagner R., Bajaj A. (2023). Full-endoscopic spine surgery diminishes surgical site infections—A propensity score-matched analysis. Spine J..

[23] Fraser R.D., Osti O.L., Vernon-Roberts B. (1987). Discitis after discography. J. Bone Jt. Surg. Br..

[24] Fraser R.D. (1984). Chymopapain for the treatment of intervertebral disc herniation. The final report of a double-blind study. Spine.

[25] Santhanam R., Lakshmi K. (2015). A Retrospective Analysis of the Management of Postoperative Discitis: A Single Institutional Experience. Asian Spine J..

[26] Jain M., Sahu R.N., Gantaguru A., Das S.S., Tripathy S.K., Pattnaik A. (2019). Postoperative Lumbar Pyogenic Spondylodiscitis: An Institutional Review. J. Neurosci. Rural Pract..

[27] Basu S., Ghosh J.D., Malik F.H., Tikoo A. (2012). Postoperative discitis following single-level lumbar discectomy: Our experience of 17 cases. Indian J. Orthop..

[28] Fraser R.D., Osti O.L., Vernon-Roberts B. (1989). Iatrogenic discitis: The role of intravenous antibiotics in prevention and treatment. An experimental study. Spine.

[29] Hadjipavlou A.G., Mader J.T., Necessary J.T., Muffoletto A.J. (2000). Hematogenous pyogenic spinal infections and their surgical management. Spine.

[30] Meyer B., Schaller K., Rohde V., Hassler W. (1995). The C-reactive protein for detection of early infections after lumbar microdiscectomy. Acta Neurochir..

[31] Pull ter Gunne A.F., Mohamed A.S., Skolasky R.L., van Laarhoven C.J., Cohen D.B. (2010). The presentation, incidence, etiology, and treatment of surgical site infections after spinal surgery. Spine.

[32] Gouliouris T., Aliyu S.H., Brown N.M. (2010). Spondylodiscitis: Update on diagnosis and management. J. Antimicrob. Chemother..

[33] Svensson O., Parment P.A., Blomgren G. (1987). Orthopaedic infections by Serratia marcescens: A report of seven cases. Scand. J. Infect. Dis..

[34] Warren N.P., Coombs R.R. (1991). Delayed Serratia marcescens osteomyelitis following a gunshot injury. Injury.

[35] Bouza E., García de la Torre M., Erice A., Cercenado E., Loza E., Rodríguez-Créixems M. (1987). Serratia bacteremia. Diagn. Microbiol. Infect. Dis..

[36] Yu W.L., Lin C.W., Wang D.Y. (1998). Serratia marcescens bacteremia: Clinical features and antimicrobial susceptibilities of the isolates. J. Microbiol. Immunol. Infect..

[37] Hadjipavlou A.G., Gaitanis I.N., Papadopoulos C.A., Katonis P.G., Kontakis G.M. (2002). Serratia spondylodiscitis after elective lumbar spine surgery: A report of two cases. Spine.

[38] Liebergall M., Chaimsky G., Lowe J., Robin G.C., Floman Y. (1991). Pyogenic vertebral osteomyelitis with paralysis: Prognosis and treatment. Clin. Orthop. Relat. Res..

[39] Santiago K., Cheng J., Jivanelli B., Lutz G. (2021). Infections Following Interventional Spine Procedures: A Systematic Review. Pain Physician.

[40] Hartog A. (2010). Interventional treatment for low back pain: General risks. Phys. Med. Rehabil. Clin. N. Am..

[41] Nelson A.M., Nagpal G. (2018). Interventional Approaches to Low Back Pain. Clin. Spine Surg..

[42] Ritter J.M., Muehlenbachs A., Blau D.M., Paddock C.D., Shieh W.-J., Drew C.P., Batten B.C., Bartlett J.H., Metcalfe M.G., Pham C.D. (2013). Exserohilum infections associated with contaminated steroid injections: A clinicopathologic review of 40 cases. Am. J. Pathol..

[43] Smith R.M., Schaefer M.K., Kainer M.A., Wise M., Finks J., Duwve J., Fontaine E., Chu A., Carothers B., Reilly A. (2013). Fungal infections associated with contaminated methylprednisolone injections. N. Engl. J. Med..

[44] Lohse N., Obel N. (2002). Meningitis caused by herpes simplex virus type 2. Ugeskr Laeger.

[45] Lee J.W., Lee E., Lee G.Y., Kang Y., Ahn J.M., Kang H.S. (2018). Epidural steroid injection-related events requiring hospitalisation or emergency room visits among 52,935 procedures performed at a single centre. Eur. Radiol..

[46] Kim B.R., Lee J.W., Lee E., Kang Y., Ahn J.M., Kang H.S. (2020). Intra-articular facet joint steroid injection-related adverse events encountered during 11,980 procedures. Eur. Radiol..

[47] Bosnak V.K., Karaoglan I., Erkutlu I., Namiduru M. (2017). Nosocomial spondylodiscitis after intradiscal electrothermal therapy: Case series. J. Pak. Med. Assoc..

[48] Dall B.E., Rowe D.E., Odette W.G., Batts D.H. (1987). Postoperative discitis. Diagnosis and management. Clin. Orthop. Relat. Res..

[49] Postacchini F., Cinotti G., Perugia D. (1993). Post-operative intervertebral discitis. Evaluation of 12 cases and study of ESR in the normal postoperative period. Ital. J. Orthop. Traumatol..

[50] Klinger M., Driesen W., Brock M., Klinger M. (1982). Spondylitis—A Complication Following Lumbar Disc Operations. Computerized Tomography Brain Metabolism Spinal Injuries.

[51] Rohde V., Meyer B., Schaller C., Hassler W.E. (1998). Spondylodiscitis after lumbar discectomy. Incidence and a proposal for prophylaxis. Spine.

[52] Singh D.K., Singh N., Das P.K., Malviya D. (2018). Management of Postoperative Discitis: A Review of 31 Patients. Asian J. Neurosurg..

[53] Ahsan K., Hasan S., Khan S.I., Zaman N., Almasri S.S., Ahmed N., Chaurasia B. (2020). Conservative versus operative management of postoperative lumbar discitis. J. Craniovertebr. Junction Spine.

[54] Ahsan M.K., Hasan M.S., Khan M.S.I., Sakeb N. (2021). Management of post-operative discitis following discectomy in a tertiary-level hospital. J. Orthop. Surg..

[55] Deyo R.A., Cherkin D.C., Loeser J.D., Bigos S.J., Ciol M.A. (1992). Morbidity and mortality in association with operations on the lumbar spine. The influence of age, diagnosis, and procedure. J. Bone Jt. Surg. Am..

[56] Rawlings C.E., Wilkins R.H., Gallis H.A., Goldner J.L., Francis R. (1983). Postoperative intervertebral disc space infection. Neurosurgery.

[57] Kang B.U., Lee S.H., Ahn Y., Choi W.C., Choi Y.G. (2010). Surgical site infection in spinal surgery: Detection and management based on serial C-reactive protein measurements. J. Neurosurg. Spine.

[58] Dowdell J., Brochin R., Kim J., Overley S., Oren J., Freedman B., Cho S. (2018). Postoperative Spine Infection: Diagnosis and Management. Glob. Spine J..

[59] Zakaria Mohamad Z., ARahim A., Kow R.Y., Karupiah R.K., Zainal Abidin N.A., Mohamad F. (2022). Diagnostic Accuracy and Adequacy of Computed Tomography Versus Fluoroscopy-Guided Percutaneous Transpedicular Biopsy of Spinal Lesions. Cureus.

[60] Sertic M., Parkes L., Mattiassi S., Pritzker K., Gardam M., Murphy K. (2019). The Efficacy of Computed Tomography-Guided Percutaneous Spine Biopsies in Determining a Causative Organism in Cases of Suspected Infection: A Systematic Review. Can. Assoc. Radiol. J..

[61] Marschall J., Bhavan K.P., Olsen M.A., Fraser V.J., Wright N.M., Warren D.K. (2011). The impact of prebiopsy antibiotics on pathogen recovery in hematogenous vertebral osteomyelitis. Clin. Infect. Dis..

[62] Duarte R.M., Vaccaro A.R. (2013). Spinal infection: State of the art and management algorithm. Eur. Spine J..

[63] Garcia-Vidal C., Cabellos C., Ayats J., Font F., Ferran E., Fernandez-Viladrich P. (2009). Fungal postoperative spondylodiscitis due to *Scedosporium prolificans*. Spine J..

[64] Zou M.-X., Peng A.-B., Dai Z.-H., Wang X.-B., Li J., Lv G.-H., Deng Y.-W., Wang B. (2015). Postoperative initial single fungal discitis progressively spreading to adjacent multiple segments after lumbar discectomy. Clin. Neurol. Neurosurg..

[65] Tsantes A.G., Papadopoulos D.V., Vrioni G., Sioutis S., Sapkas G., Benzakour A., Benzakour T., Angelini A., Ruggieri P., Mavrogenis A.F. (2020). Spinal Infections: An Update. Microorganisms.

[66] Paez D., Sathekge M.M., Douis H., Giammarile F., Fatima S., Dhal A., Puri S.K., Erba P.A., Lazzeri E., Ferrando R. (2021). Comparison of MRI, [^18^F]FDG PET/CT, and ^99m^Tc-UBI 29-41 scintigraphy for postoperative spondylodiscitis—A prospective multicenter study. Eur. J. Nucl. Med. Mol. Imaging.

[67] Van Goethem J.W., Parizel P.M., van den Hauwe L., Van de Kelft E., Verlooy J., De Schepper A.M. (2000). The value of MRI in the diagnosis of postoperative spondylodiscitis. Neuroradiology.

[68] Levi A.D., Dickman C.A., Sonntag V.K. (1997). Management of postoperative infections after spinal instrumentation. J. Neurosurg..

[69] Stambough J.L., Beringer D. (1992). Postoperative wound infections complicating adult spine surgery. J. Spinal Disord..

[70] Rayes M., Colen C.B., Bahgat D.A., Higashida T., Guthikonda M., Rengachary S., Eltahawy H.A. (2010). Safety of instrumentation in patients with spinal infection. J. Neurosurg. Spine.

[71] Lee M.C., Wang M.Y., Fessler R.G., Liauw J., Kim D.H. (2004). Instrumentation in patients with spinal infection. Neurosurg. Focus.

[72] Turel M.K., Kerolus M., Deutsch H. (2017). The role of minimally invasive spine surgery in the management of pyogenic spinal discitis. J. Craniovertebr. Junction Spine.

[73] Hadjipavlou A.G., Katonis P.K., Gaitanis I.N., Muffoletto A.J., Tzermiadianos M.N., Crow W. (2004). Percutaneous transpedicular discectomy and drainage in pyogenic spondylodiscitis. Eur. Spine J..

[74] Ito M., Abumi K., Kotani Y., Kadoya K., Minami A. (2007). Clinical outcome of posterolateral endoscopic surgery for pyogenic spondylodiscitis: Results of 15 patients with serious comorbid conditions. Spine.

[75] Onik G., Shang Y.L., Maroon J.C. (1990). Automated percutaneous biopsy in postoperative diskitis: A new method. AJNR Am. J. Neuroradiol..

[76] Nagata K., Ohashi T., Ariyoshi M., Sonoda K., Imoto H., Inoue A. (1998). Percutaneous suction aspiration and drainage for pyogenic spondylitis. Spine.

[77] Yu C.H. (2020). Full-endoscopic debridement and drainage treating spine infection and psoas muscle abscess. J. Spine Surg..

[78] Lin G.-X., Kim J.-S., Sharma S., Sun L.-W., Wu H.-H., Chang K.-S., Chen Y.-C., Chen C.-M. (2019). Full Endoscopic Discectomy, Debridement, and Drainage for High-Risk Patients with Spondylodiscitis. World Neurosurg..

[79] Choi E.J., Kim S.Y., Kim H.G., Shon H.S., Kim T.K., Kim K.H. (2017). Percutaneous Endoscopic Debridement and Drainage with Four Different Approach Methods for the Treatment of Spinal Infection. Pain Physician.

[80] Hagel V., Dymel F., Werle S., Barrera V., Farshad M. (2023). Combined endoscopic and microsurgical approach for the drainage of a multisegmental thoracolumbar epidural abscess: Illustrative case. J. Neurosurg. Case Lessons.

[81] Hadjipavlou A.G., Korovessis P.G., Kakavelakis K.N., Vaccaro A.R., Eck J.C. (2010). Spine infections: Medical versus surgical treatment options. Controversies in Spine Surgery.

[82] Arya S., Crow W.N., Hadjipavlou A.G., Nauta H.J., Borowski A.M., Vierra L.A., Walser E. (1996). Percutaneous transpedicular management of discitis. J. Vasc. Interv. Radiol..

[83] Borowski A.M., Crow W.N., Hadjipavlou A.G., Chaljub G., Mader J., Cesani F., Vansonnenberg E., Borowski W.N.C.A.M., Stevens M., Helms C. (1998). Interventional radiology case conference: The University of Texas Medical Branch. Percutaneous management of pyogenic spondylodiskitis. AJR Am. J. Roentgenol..

[84] McCain G.A., Harth M., Bell D.A., Disney T.F., Austin T., Ralph E. (1981). Septic Discitis. J. Rheumatol..

[85] Hadjipavlou A.G., Crow W.N., Borowski A., Mader J.T., Adesokan A., Jensen R.E. (1998). Percutaneous transpedicular discectomy and drainage in pyogenic spondylodiscitis. Am. J. Orthop..

